# Novel insights from spatial transcriptome analysis in solid tumors

**DOI:** 10.7150/ijbs.83098

**Published:** 2023-09-04

**Authors:** Jun Du, Zhi-Jie An, Zou-Fang Huang, Yu-Chen Yang, Ming-Hui Zhang, Xue-Hang Fu, Wei-Yang Shi, Jian Hou

**Affiliations:** 1 Department of Hematology, Renji Hospital, School of Medicine, Shanghai Jiao Tong University, 160 Pujiang Road, Shanghai, 200127, China.; 2 School of Medicine, Shanghai Jiao Tong University, Shanghai, 200025, China.; 3 Ganzhou Key Laboratory of Hematology, Department of Hematology, The First Affiliated Hospital of Gannan Medical University, Ganzhou, Jiangxi, 341000, China.; 4 Ministry of Education Key Laboratory of Marine Genetics and Breeding, College of Marine Life Sciences, Ocean University of China, Qingdao 266003, China.

**Keywords:** spatial transcriptomics, solid tumor, tumor heterogeneity, tumor microenvironment

## Abstract

Since its first application in 2016, spatial transcriptomics has become a rapidly evolving technology in recent years. Spatial transcriptomics enables transcriptomic data to be acquired from intact tissue sections and provides spatial distribution information and remedies the disadvantage of single-cell RNA sequencing (scRNA-seq), whose data lack spatially resolved information. Presently, spatial transcriptomics has been widely applied to various tissue types, especially for the study of tumor heterogeneity. In this review, we provide a summary of the research progress in utilizing spatial transcriptomics to investigate tumor heterogeneity and the microenvironment with a focus on solid tumors. We summarize the research breakthroughs in various fields and perspectives due to the application of spatial transcriptomics, including cell clustering and interaction, cellular metabolism, gene expression, immune cell programs and combination with other techniques. As a combination of multiple transcriptomics, single-cell multiomics shows its superiority and validity in single-cell analysis. We also discuss the application prospect of single-cell multiomics, and we believe that with the progress of data integration from various transcriptomics, a multilayered subcellular landscape will be revealed.

## 1. Introduction

Spatial and temporal dynamics are closely related to all the properties of a cell life cycle, including cytogenesis, differentiation, function, and death 1. To explore the spatial and temporal features of cells, spatiotemporal transcriptomics was created, which includes spatial transcriptomics and temporal transcriptomics. Spatiotemporal transcriptomics provides a platform for researchers to analyze the genome and transcriptome at a subcellular atlas and comprehend the relative position of cells in tissue and the interaction with neighbors that could explain the function of cells.

The popularization of single-cell RNA sequencing (scRNA-seq) 2 ushered in the era of single-cell analysis and enabled researchers to explore the identities of diverse cell types in complex tissue at the single-cell level. ScRNA-seq is based on high-throughput sequencing, also known as next-generation sequencing (NGS), which is used for detecting the single-cell transcriptome and providing high-resolution views of intercellular heterogeneity, revealing the precise location of transcription boundaries with a single-base resolution 2, and it can provide the differentiation trajectory of cells.

In the workflow of scRNA-seq, the preparation of a single-cell suspension is indispensable for sequencing; however, this results in the loss of spatial information for the cells within the tissue. The spatial distribution of distinct cell types is a key factor in helping researchers comprehend the biological function and pathological changes of tissue. Especially in the study of tumor pathology, a spatially resolved cell atlas reveals a distinct border of tumor-infiltrated areas and stroma, cellular interaction between tumor and normal cells and a molecular-morphological map of the tumor.

To correlate cell type information with their spatial location within the tissue, a new technique termed spatial transcriptomics (ST) was first developed in 2016 by Ståhl et al., which implements the visualization and quantitative analysis of intact tissue with spatially resolved transcriptome data 3. The original ST approaches do not provide single-cell level resolution 4. Since then, the combination of scRNA-seq and ST 5-7, which both retain spatial distribution and single-cell resolution, has become the cornerstone of cellular heterogeneity studies in complex tissues.

This article reviews the recent advances in solid tumor tissue analysis by ST and summarizes the utilization of ST from different perspectives, including cell clustering and interaction, cellular metabolism, gene expression, immune cell programs and combination with other techniques. In recent years, single-cell multimodal omics have been developed rapidly, which is another major step toward comprehension of the inner workings, network connectivity and operational principles of cells; hence, we consider the future of ST as a crucial part of single-cell multimodal omics analysis.

## 2. Origin of spatial transcriptomics and insights

### 2.1 History of spatial transcriptomics

Before the phrase “spatial transcriptomics” was coined, multiple technologies were designed to explore mRNA expression in tissue sections with spatially resolved data. Since the 1970s, *in situ* hybridization (ISH) has been conducted to visualize gene expression while retaining spatial distribution, including radioactive ISH (1969), fluorescent *in situ* hybridization (FISH) (1977), and whole-mount ISH (WM ISH) (1989). These techniques analyze gene expression on a single gene basis, while researchers anticipated a technique accomplishing the exploration of more genes or even whole transcriptomes, which eventually facilitated the emergence of ST 8.

The foundation of some ST technologies can be traced back to IR and UV laser capture microdissection (LCM) (1996) 9, which soon after was combined with microarray technology (1995) in 1999 10. Basically, present-era ST techniques are divided into five subcategories: LCM, single molecular FISH (smFISH), *in situ* sequencing (ISS), *in situ* array capturing (Array) and in silico reconstruction of spatial data 11, whose representative methods are GeoMX Digital Spatial Profiler (DSP), multiplexed error-robust FISH (MERFISH), Fluorescent *In Situ* Sequencing (FISSEQ), 10x Genomics Visum and microfluidic Deterministic Barcoding in Tissue for spatial omics sequencing (DBiT-seq).

Currently, ST has already been used on various human tissues, especially in solid tumors, and the combination of scRNA-seq and ST, which both retain the spatial distribution and single-cell resolution, has become the cornerstone in tissue section analysis. Strictly, the single-cell resolution requires spatial chips with subcellular resolution; otherwise, present low-resolution ST can only use single-cell data for deconvolution conjecture. In particular, GeoMX DSP, which is based on the laser region of interest (laser ROI), can reach a subcellular resolution. With the combination of scRNA-seq, GeoMX DSP was performed on COVID-19 tissues and provided critical insights into the pathogenesis of severe COVID-19 12.

### 2.2 Commonly applied ST technologies

Since the initial proposal in 2016, diverse spatial transcriptomics techniques utilizing distinct strategies have gained rapid and widespread adopted. Each of these methods possesses unique features, accompanied by their respective strengths and limitations. In this section, we expound upon the fundamental principles of the two most commonly utilized commercialized ST technologies: the 10x Genomics Visium and GeoMX DSP platforms.

#### 2.2.1 10x Genomics Visium

10x Genomics Visium epitomizes a cutting-edge platform that leverages comprehensive measurement of mRNA in tissue sections while concurrently capturing the spatial resolved data for precise positioning.

The Visium Spatial Gene Expression Slide is consisted with four 6.5 x 6.5 mm capturing area, which contains approximately 5000 spots with millions of capture probes in each spot 13. Following tissue section permeabilization, cellular mRNA is made accessible and transformed into complementary DNA (cDNA) with spatial barcodes through reverse transcription 13. The incorporation of spatial barcodes allows for the precise identification of each mRNA transcript's location within the tissue section. By constructing a spatial gene expression library and performing RNA sequencing, researchers are able to analyze mRNA expression profiles within intact tissue sections while also visualizing them.

The compatible tissue type of 10x Genomics Visium includes formalin-fixed, paraffin-embedded (FFPE) tissue and fresh frozen tissue samples. While the 10x Genomics Visium platform boasts an impressive resolution of 1-10 cells on average with a diameter of 55µm per spot, it falls short of achieving single-cell level resolution, which is the main drawback of this technique. 10x Genomics Visium is considered a highly advanced ST technology that enables researchers to comprehensively analyze the whole transcriptome with a panoramic view of the section.

#### 2.2.2 GeoMX DSP

GeoMX DSP quantified mRNA transcripts and protein expression using indexing oligonucleotides that are attached to mRNA capture probes or antibodies 14. After incubating the sample with oligonucleotide tags and visualizing it using fluorescent markers, the researchers were able to identify the Regions of Interest (ROI) - specific areas for analyzing the mRNA or protein pattern. The GeoMX DSP platform utilizes a design incorporating the indexing oligonucleotides with a UV-photocleavable linker 14. When the ROI is exposed to UV light, the DSP barcodes are released, subsequently detected and sequenced. Similar to 10x Genomics Visium, the GeoMX DSP platform is compatible with both FFPE tissue and fresh frozen tissue samples.

The integration of multi-omics has long posed a challenging issue, however, the GeoMX DSP platform enables the combination of ST and spatial proteomics through simultaneous sequencing of transcriptome and proteome. With a spatial resolution that extends up to a depth of 10 µm 15, the GeoMX DSP theoretically achieved single-cell accuracy. Unlike 10x Genomics Visium, which provides a comprehensive view of the entire tissue section, the selection of ROI in this solution allows researchers to explore specific foci of interest within the tissue.

### 2.3 Insights from spatial transcriptomics

**Figure 1:**
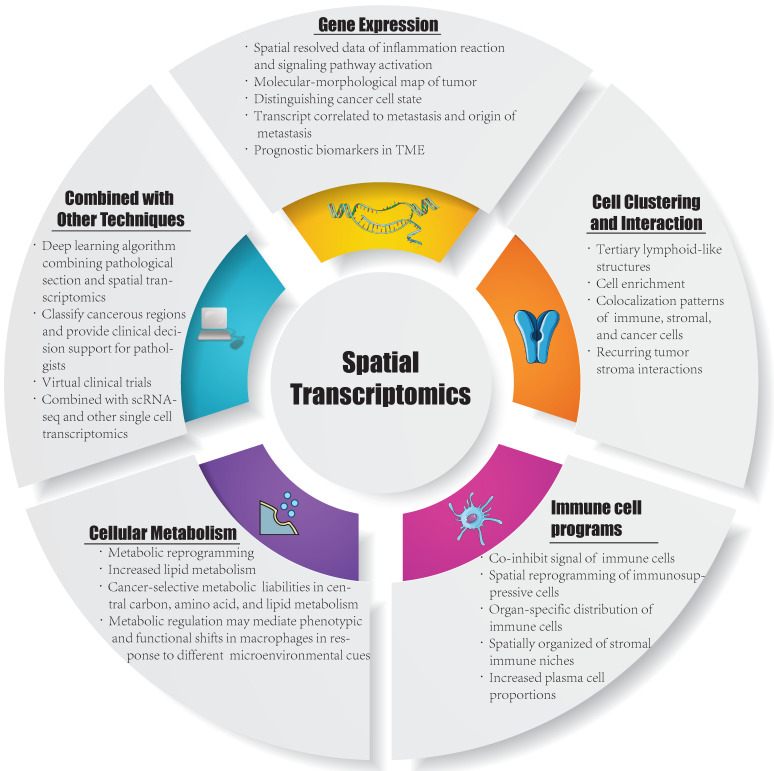
** Inspirations in spatial transcriptomics.** TME: tumor microenvironment; ScRNA-seq: single cell RNA sequencing.

**Figure 2:**
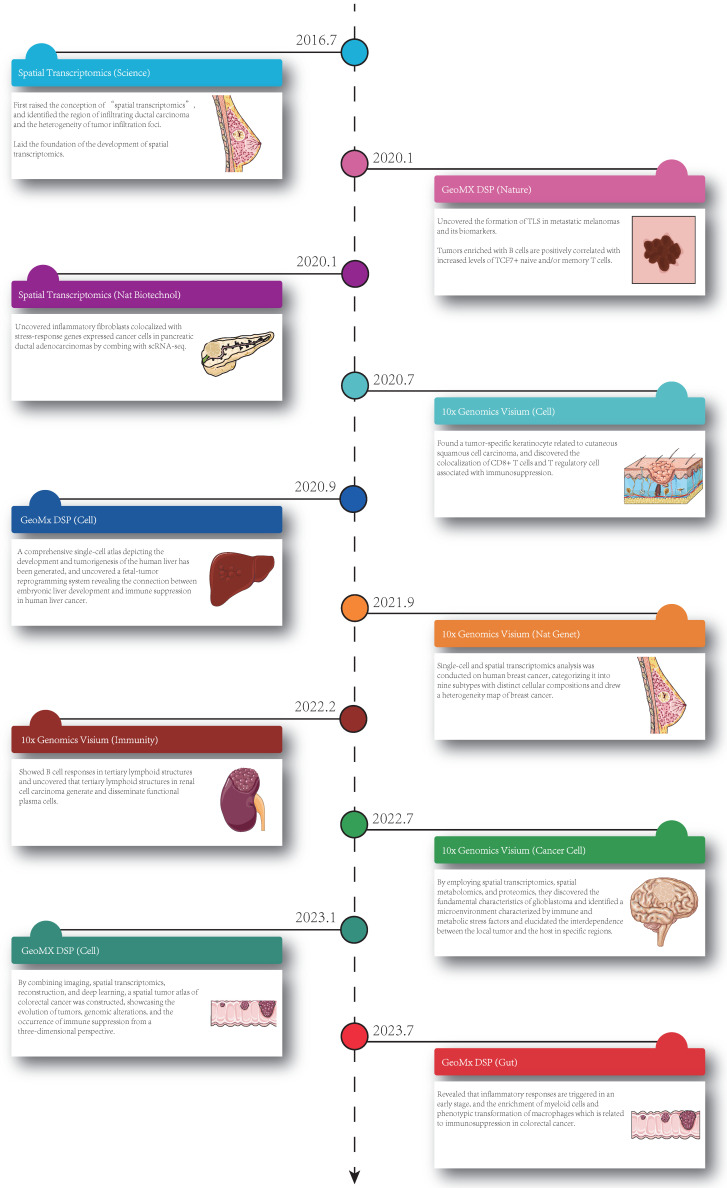
** Ten significant findings of spatial transcriptomics in cancer research.** The timeline showed the publication dates of relevant studies, the technologies, the journals and the key findings 3, 7, 16, 17, 32, 34, 46, 59-61. Nat Biotechnol: Nature Biotechnology; GeoMx DSP: GeoMX Digital Spatial Profiler; TLS: tertiary lymphoid structures; TCF: T-cell factor; ScRNA-seq: single cell RNA sequencing; CD8: cluster of differentiation 8; Nat Genet: Nature Genetics.

Currently, ST is applied in various fields, including development, neuroscience, pathology, tumorigenesis, etc., and has already led to successful outcomes in the analysis of spatial distributions of distinct cell types, and in particular, ST has been extensively performed in the analysis of solid tumor tissues. Considering this, in this section, we summarize the insight that ST brings to the study of solid tumors, including cell clustering and interaction, cellular metabolism, gene expression, immune cell programs and combination with other techniques (Figure 1). Besides, we have summarized ten significant findings of spatial transcriptomics in cancer research (Figure 2).

#### 2.3.1 Cell clustering and interaction

In the workflow of ST, cell clustering and analysis of cellular interactions are indispensable, which leads to significant outcomes in the analysis of tissue sections. With the data of cell clustering and the projection of the results on tissue images, ST provides an understanding of cell enrichment with the spatial distribution and can define new subsets of cells. For instance, Ji et al. defined a new subset of tumor-specific keratinocyte populations related to human squamous cell carcinoma by the specific expression pattern of epithelial-mesenchymal transition markers 7, and it was applied to distinguish the border of tumor infiltration and stromal-immune niches 16.

Andersson et al. showed that with the assistance of expression-based clustering, the definition of cell types and heterogeneity of tumors could be analyzed, and they found immune cells involved and the relative genes overexpressed i.e., macrophages (APOE etc.); lymphocytes (HLA class I and II); cancer cells (ErbB2; EPCAM and CDH1) 5. Moreover, ST provides insights into the colocalization of cells that allows analysis of cell-cell interactions, including inflammatory fibroblasts colocalized with stress-response genes expressed in cancer cells and colocalization of stromal, immune and cancerous cells in TME recurring tumor-stroma interactions 17-19.

#### 2.3.2 Cellular metabolism

ST is also widely performed to determine the key aspects in the processes of cellular metabolism for different spatial foci of tumors showing distinct metabolic patterns, and metabolic reprogramming is a common phenomenon in solid tumors. Combined with scRNA-seq, ST has shown superiority in the study of cellular metabolism by analyzing the expression of key transcripts in metabolism-related gene pathways. In solid tumor tissues, phenotypic and functional shifts in macrophages are related to metabolic regulation in response to changes in the microenvironment 6. Metabolism in the TME is also strongly associated with tumor formation; for instance, myeloid cells metabolizing arginine and CD4(+) T cells in neuroblastoma together create a favorable microenvironment for tumorigenesis 20.

With spatially resolved tissue data, the metabolic affinities of various cell subsets in the TME can be distinguished. In a cell subset termed invasive micropapillary carcinoma (IMPC) of breast cancer, higher lipid metabolism is found in all IMPC clusters and metabolic disturbance IMPC subpopulations located in different spatial areas 21. Wang et al. modeled the network of metabolism in prostate cancer and discovered that prostaglandin metabolism genes were expressed at higher levels in tumor tissue than in adjacent tissue, and they demonstrated the unique liabilities of metabolism in the TME 22.

#### 2.3.3 Gene expression

Another crucial application of ST is the quantitative analysis of gene expression with the knowledge of spatial distribution in intact tissue. Before the large-scale application of ST, researchers could hardly describe the consistent, gene expression patterns and cellular interactions in different regions of the TME, which impeded the comprehension of tumor heterogeneity. Moreover, the alteration of gene expression is a significant aspect in tumor progression; without ST, the analysis of transcriptomic changes is limited to single cell types rather than the overall analysis of distinct spatial regions of the TME. In studies of solid tumors, the heterogeneity of the TME shows strategic significance in tumorigenesis, progression, metastasis and the selection of therapy for tumor treatment.

With the advantage of retaining spatially resolved data at the single-cell level with scRNA-seq integration, ST provides a platform for researchers to explore the differential genes among various constituents, the traits of gene expression with spatial distribution in the TME 23, key transcriptomic changes and copy number variation (CNV) events 24 and the biomarkers associated with prognosis in the TME with spatial coordinates 25.

In colorectal cancer, Wang et al. uncovered increased PD-L1 expression at the transcriptional and translational levels in patients receiving immunotherapy compared with chemotherapy, which was considered feasible for the exploration of prognostic markers with spatially resolved data in the TME 25. With the clarification of the tumor-infiltrated area, ST showed superiority in delineating the extent of cancer foci more accurately 26.

The spatial distribution resolved data also provides insights into the visualization of inflammatory reactions 5 and the analysis of prolonged interferon signaling activation 24 (with scRNA-seq integration). Moreover, the application of ST in solid tumors reveals the whole process of tumor development, for instance, fetal-like reprogramming and signaling pathways related to maintaining an onco-fetal ecosystem in tumorigenesis, the emergence of stress-like subsets since the preliminary phases of oncogenesis express a set of genes and show properties of drug resistance 27, epithelial-mesenchymal transition and angiogenesis 7, 28 polyclonal origins of metastasis 29 and transcripts correlated with metastasis 30, 31.

#### 2.3.4 Immune cell programs

Immune dysregulation in the TME plays a significant role in tumor growth, metastasis and immunosuppression. To ameliorate immune disorders and reverse immune suppression in the TME, researchers must understand the mechanisms of immunoregulation in tumor-infiltrated and normal areas as well as the spatial distribution of immune cells organized in tumors. Through its integration with scRNA-seq data, ST has been used for spatially mapping tertiary lymphoid-like structures, and revealing the specific interferon response in a particular region in a section, that type I interferon was associated with the coupling between particular T cell and macrophage states and B and T cells were co-localized in some patients 5. Furthermore, the composition and organization of the TME, including the presence of tertiary lymphoid-like structures, are additional factors that can serve as predictors of the clinical outcomes of immunotherapy 32. The leading edge of the immune reaction and the stromal-immune niches are marked with spatially resolved data from ST, which provide insights into immune regulation in antitumor therapies 16.

Immunosuppression is acknowledged as the mediator of escape from immune system activation, and to comprehend the spatial reprogramming of immunosuppressive cells in the TME, ST is necessary. Combined with scRNA-seq, multiple cell types participating in immunosuppression were observed in squamous cell carcinoma, including coinhibitory signals on DCs and exhausted T cells and recruitment of regulatory T cells (Tregs) 7.

#### 2.3.5 Combination with other techniques

With the rapid development of genomics, transcriptomics, proteomics tools, etc., the application of multiomics has already become the trend in tumor tissue analysis. ST, which enables researchers to perform quantitative analysis and retains the spatial distribution of tissue sections, is a crucial part of the exploration of spatial heterogeneity in the TME. In addition, since ST was first reported, the combination of ST and scRNA-seq has been the most popular technique in normal and tumor tissue sequencing.

While ST technology continues to evolve, another accelerated developing technique is spatial proteomics, and researchers are engaged in the combined application of ST and spatial proteomics, for the levels of RNA transcription in ST cannot directly reflex the levels of protein expression and spatial proteomics supplements this deficiency. Presently, Multiplexed Ion Beam Imaging Time-of-flight Mass Spectrometry (MIBI-TOF) which is based on metal labeled antibodies imaged by orthogonal time-of-flight mass spectrometry 33 was incorporated with ST and scRNA-seq in human squamous cell carcinoma to image the expression of and protein markers in the tumor, stroma and immune cells, and analyze the immunodepression pattern detected in ST and scRNA-seq 7. Moreover, GeoMx DSP, based on identifying the releasing of oligonucleotide tags, enables simultaneous analysis of both RNA transcriptome and protein expression in a single tissue section 14. This innovative technology has already been extensively utilized in various types of tumors.

Spatial metabolomics, along with ST and spatial proteomics, plays a critical role in enabling spatially resolved multi-omics analysis. By integrating spatially resolved multi-omics, Ravi VM et al. spatially visualize the expression pattern of genes by ST and spatial proteomics and uncovered the mechanisms of dynamic adaptation of glioblastoma with the application of spatial metabolomics for metabolic factors is crucial in the dynamic adaptation of glioblastoma 34.

Spurred by advances in computer hardware and software, computers are currently widely used in various disciplines, particularly the combination of mathematics and computer science, which was developed to solve the challenge of building statistical models from massive datasets and termed machine learning 35. A deep learning algorithm combining pathological sections and spatial transcriptomics provides insights into the accurate prediction of spatially variable genes and the capture of high-resolution gene expression heterogeneity 36. Applying convolutional neural networks (CNN) for spatial heterogeneity in gene expression of tissue analysis, a deep-learning algorithm was developed by He et al. on a new ST dataset in 68 tissue sections from 23 breast cancer patients to predict genes with the highest mean expression and the spatial variable genes, and the test showed its accuracy, which was named ST-Net and combined histology examination with ST 36. Additionally, it has been used for the recognition of tumor-infiltrated regions in entire tissue sections automatically, by which clinical decisions could be furnished to pathologists in the future 37.

## 3. Advances in spatial transcriptomics in solid tumors

The tumor microenvironment (TME), the embodiment of heterogeneity, is closely related to tumorigenesis, progression and metastasis. Once we attribute tumorigenesis to mutationally corrupted cancer cells, studies have already proven that TME also plays a crucial role in tumorigenesis. Moreover, heterogeneity, which plays a critical role in drug resistance, has become a tremendous challenge in antitumor treatments. To analyze the TME and tumor heterogeneity, ST has emerged as a powerful tool to obtain the biological properties of different cell types and cell interactions with spatially resolved information 25. With the development of ST, it has been used in multiple types of solid tumor tissue, including lung cancer, breast cancer, gastrointestinal cancer, and prostate cancer (Table 1).

### 3.1 Lung cancer

Lung cancer has already become the leading cause of mortality in both sexes worldwide. Presently, multiple therapies for lung cancer, including surgery, radiotherapy, chemotherapy, immunotherapy, etc., and the emergence of immunotherapy, especially PD-1/PD-L1 blockade, have shown superiority. However, the effect of immunotherapy varies in different patients, and ST provides insight into tumor heterogeneity with spatially resolved datasets, which may explain and predict the prognosis of various patients after immunotherapy.

Jon et al. conducted research on non-small cell lung cancer (NSCLC) patients to investigate the biomarkers linked to advantageous PD-1 checkpoint blockage using the GeoMx DSP, and they showed the potential of DSP in identifying spatially informative biomarkers of the PD-1 checkpoint blockade response in NSCLC and confirmed alternative immune predictors with spatial context deserving larger independent cohorts' validation 38. By applying ST to detect the composition of the TME, they revealed how distinct components of the TME determine the outcome of PD-1 checkpoint blockade.

Moreover, Larroquette et al. performed ST on 16 NSCLC tumors using DSP to determine NSCLC cells with different expression levels of CD163+ and to explore the determining factors that affect the effect of immune checkpoint blockers in tumors with increasing CD163+ expression, and they found that tumors with high CD163+ cell infiltration showed upregulation of ITGAM, CD27, and CCL5 39. Improved outcomes of immunotherapy were revealed to be related to the increased expression of genes including the M1 phenotype and interferon-γ signaling pathway in high macrophage infiltration tumors, and they found upregulation of CSF1R in responders 39.

### 3.2 Breast cancer

Breast cancer is a heterogeneous disease that develops due to a combination of genetic and environmental factors 40. The complex architecture and the specific spatial distribution of assorted cell types are intimately linked to tumor progression and response to therapy. Understanding tumor heterogeneity plays a critical role in clarifying prognoses and providing implications for chemotherapies for breast cancer.

Lv et al. drew transcriptomic maps of invasive micropapillary carcinoma and revealed its extensive heterogeneity and discovered that the stromal areas displayed different gene expression patterns with the ST platform of 10x Genomics Visium (Visium) 21. Furthermore, the curative effect of chemotherapeutic drugs is pertinent to the spatial distribution of pharmacogenes. Powell et al. applied Visium spatial transcriptomics to breast tumor tissue and revealed that the heterogeneity of pharmacogene expression may have significant implications for cancer treatment because of the influence on drug distribution and efficacy 41.

ST incorporating various transcriptomics, especially scRNA-seq, single-nucleus RNA sequencing (snRNA-seq) and proteomics, may provide researchers with a broader range of ST applications. Andersson et al. used spatial transcriptomics (Visium) and integrated single-cell data to investigate gene expression patterns with spatially resolved data in HER-2-positive breast tumors and found a tertiary lymphoid-like structure and the colocalization of the type I interferon response with macrophages and T cells 5. He et al. combined spatial transcriptomics (Visium) with histology images and identified spatially resolved gene expression data of 102 genes and the simultaneous presence of immune activation and tumor development 36.

### 3.3 Liver cancer

Globally, liver cancer is one of the most common cancers and was the sixth most diagnosed among all sites of cancer and the fourth most common cause of mortality in 2018, and the incidence and mortality showed a sex difference, with males being at high risk for liver cancer 42. To analyze the heterogeneity of the TME with spatial data, researchers have already applied ST in liver cancer and obtained certain results.

Wu et al. performed ST on 21 tissues from seven patients with primary liver cancers based on Visium to investigate the gene expression states in different foci of the TME, and they revealed the relationship between the remodeling of the TME and tumor metastasis and the distribution of PROM1(+) and CD47(+) cancer stem cells 43. In addition, they uncovered a TLS precisely by proposing the TLS-50 signature 43. Additionally, based on the platform of Visium, Wang et al. identified the malignant subsets of the TME of hepatocellular carcinoma by hierarchical clustering, and they discovered the enrichment of CCL15 in the core region of the tumor that participated in the construction of an immunosuppressive microenvironment, which is also a molecular sign indicating poor prognosis together with CD163^44^.

In combination with scRNA-seq, Sharma et al. applied ST (GeoMx DSP) to hepatocellular carcinoma and uncovered the oncofetal reprogramming of the tumor ecosystem in which VEGF and NOTCH signaling played a significant role 45.

### 3.4 Gastrointestinal cancer

Gastric cancer is the fifth-highest diagnosed cancer in both sexes and the third-highest cause of death in all cancers, especially in males, while colorectal cancer is the third-highest in incidence and the second-highest in mortality both in males and females 42. However, clinically, spatial transcriptomics has not been widely used in defining heterogeneity within gastrointestinal cancers, in which research is urgently needed.

Kumar et al. combined ST (GeoMx DSP), bulk RNA-seq cohorts for quadrature validation, and confirmed the results with *in vitro* and vivo models and provided a high-resolution molecular resource of within-patient and between-patient pedigree status across different gastric cancer subtypes 46.

Wang et al. performed ST (GeoMx DSP) on tissue sections from patients treated by only neoadjuvant chemotherapy or in conjunction with immunotherapy, which profiled both the mRNA and protein levels and uncovered significant immune infiltration at tumor areas linked to immunotherapy treatment 25. Additionally, Wu et al. sequenced 97 samples of liver metastasis patients with colorectal cancer by scRNA-seq and ST (Visium) and discovered that immunosuppressive cells in the metastatic microenvironment experienced prominent spatial reorganization 6.

### 3.5 Pancreatic cancer

Pancreatic cancer has the highest mortality rate of all cancers, with a 5-year survival rate that has not improved since the 1960s in the United States, which is only approximately 10% 47, 48. In addition, most patients with pancreatic cancer seek clinical advice at an advanced stage due to its unique anatomical position, and overall, the prognosis of pancreatic cancer is poor.

Thus, in recent years, researchers have already begun to investigate pancreatic cancer with the assistance of scRNA-seq and ST and brought insights in diagnoses, analysis of heterogeneity and therapy of pancreatic cancer. Young et al. identified the metastasis-like primary (MLP)-1 subtype as a phenotype with extensive and powerful activation of immune-related genes applying the DSP platform 49. This study provides the basis for future precision immunotherapy studies for MLP-1 patients. Moncada et al. proposed a multimodal intersection analysis combining scRNA-seq and a microarray-based spatial transcriptomics method (Visium) to study the heterogeneity of the TME of pancreatic cancer 17. They described the spatial distribution of distinct cell types and subpopulations and showed distinct co-enrichments between different cell types 17.

With the combination of single-nucleus RNA sequencing, Hwang et al. performed ST (DSP) on 43 primary pancreatic ductal adenocarcinoma (PDAC) tumor specimens, and they identified recurring expression patterns in fibroblasts and cancerous cells and uncovered three multicellular colonies constituted by a variety of cell subtypes 50.

### 3.6 Bladder cancer

Bladder cancer is the most diagnosed malignant tumor in the urinary system, and there were 549,393 new cases in 2018, accounting for 3.0% of all types of cancer and causing 199,922 deaths in the same year 42. The postponement of diagnosis, especially for females, is the most significant factor in the mortality of bladder cancer 51.

Compared to the aforementioned tumors, bladder cancer has not been extensively addressed by ST. In one case, Gouin et al. incorporated ST (Visium) with single-nucleus RNA-seq and spatial proteomic analysis on bladder cancer and classified an epithelial subtype (expressing Cadherin 12, catenin, and other epithelial markers), which was closely related to the therapeutic response in which Cadherin 12-enriched tumors showed a superior reaction to immune checkpoint therapy compared with neoadjuvant chemotherapy 52. This study shows the potency of ST in the analysis of intact tissue from human bladder cancer, and the approach of multimodal omics warrants further study.

### 3.7 Prostate cancer

Prostate cancer, the second most common cancer in males and the fifth leading cause of death of all cancers 53, is a disease mostly occurring in aging males. However, the classification of prostate cancer varies from less aggressive localized to metastatic, which is incurable despite present treatment strategies 54.

Applied ST (DSP) to quantitatively analyze transcript and protein abundance with spatially resolved distribution, Brady et al. compared the heterogeneity between inter- and intratumor prostate cancer with the biological behavior of metastasis and identified homogeneity concerning tumor phenotype 55. In contrast to the declining expression of PD1/PD-L1 and CTLA4, the immune checkpoint protein B7-H3/CD276 showed abnormally high expression, especially in phenotypes with androgen receptor activation 55. By comparing tumoral and adjacent tissue by ST (Spatial Transcriptomics), Berglund et al. distinguished tumoral focus accurately with help from pathologic description, and they re-stratified the TME of prostate cancer 26. Wang et al. found high expression of the prostaglandin gene in tumors, and they discovered new targets with metabolic vulnerability by small molecular drugs using the 10x Genomics Visium^22^. Moreover, they predicted that inhibitors of SCD1 and SLCO2A1, which are fatty acid desaturases and transporters of prostaglandin, may slow tumoral progression.

## 4. Conclusion and future perspectives

Since the first application of ST in 2016, which was developed by Ståhl et al., this technology has already been widely applied in distinct fields, including neuroscience, oncology, developments of organ and plant biology. With the advantage of retaining spatial distribution when performing transcriptome sequencing, ST shows its superiority in the analysis of gene expression in distinct foci of tissue, the interactions between disparate compartments of tissues and the mechanism of drug resistance. Thus, the application of ST in the investigation of compartmentalization of the TME has become a routine strategy for researchers.

The value of spatial transcriptomics extends beyond studying the biological characteristics of tumors, as it also has implications for clinical diagnosis, treatment, and prognosis prediction in cancer research. By combining deep learning and spatial transcriptomics, it is possible to more accurately distinguish tumor-enriched regions, non-tumor regions, and tumor-infiltrated regions 36, 57, 58, thus providing a more accurate assessment of the extent of tumor infiltration and facilitating a refined classification of tumors based on gene expression. ST has the potential to enable the development of a novel pathology system known as "spatially resolved molecular pathology". Through the use of high-throughput sequencing, this system would offer significant advantages over traditional methods and could represent a major step forward in the field of molecular pathology. In addition, ST can assist clinical physicians in selecting individualized anti-tumor therapies and predicting patient prognosis by identifying specific gene expressions in the patient's tumor tissue and the three-dimensional immune structure of the TME. This approach represents a promising avenue for advancing the field of precision medicine.

Presently, ST has been widely performed to understand the heterogeneity of the TME in solid tumors, especially in breast cancer, and has already shown achievements. Conversely, in nonsolid tumors, especially tumors originating from the hematological system, the application of ST is inadequate, and we envision that ST technology will demonstrate feasibility in bone marrow tissue sections.

However, the restriction of resolution and sensitivity are still challenges for ST. To confront these technical shortcomings, two scenarios are feasible, which include improving the resolution and accuracy of ST and integration with other technologies. We anticipate the establishment of ST technology with higher resolution, and presently researchers are seeking the possibility of merging multiple omics in tissue analysis. To provide single-cell datasets, ST combined with scRNA-seq has already been widely used in the study of the TME, and to validate the quantitative analysis of gene expression, proteomics was incorporated with ST. With the development of next-generation sequencing, the era of single-cell multiomics, including and not restricted to genomics, single-cell transcriptomics, spatial transcriptomics and proteomics, is around the corner, which offers an opportunity for analyzing DNA, mRNA, and proteins at single-cell resolution. With the maturation of single-cell multiomics, we believe that we will gain deeper insights into tumorigenesis, progression and metastasis and thus propose the development of new strategies for antitumor therapies.

## Figures and Tables

**Figure 3:**
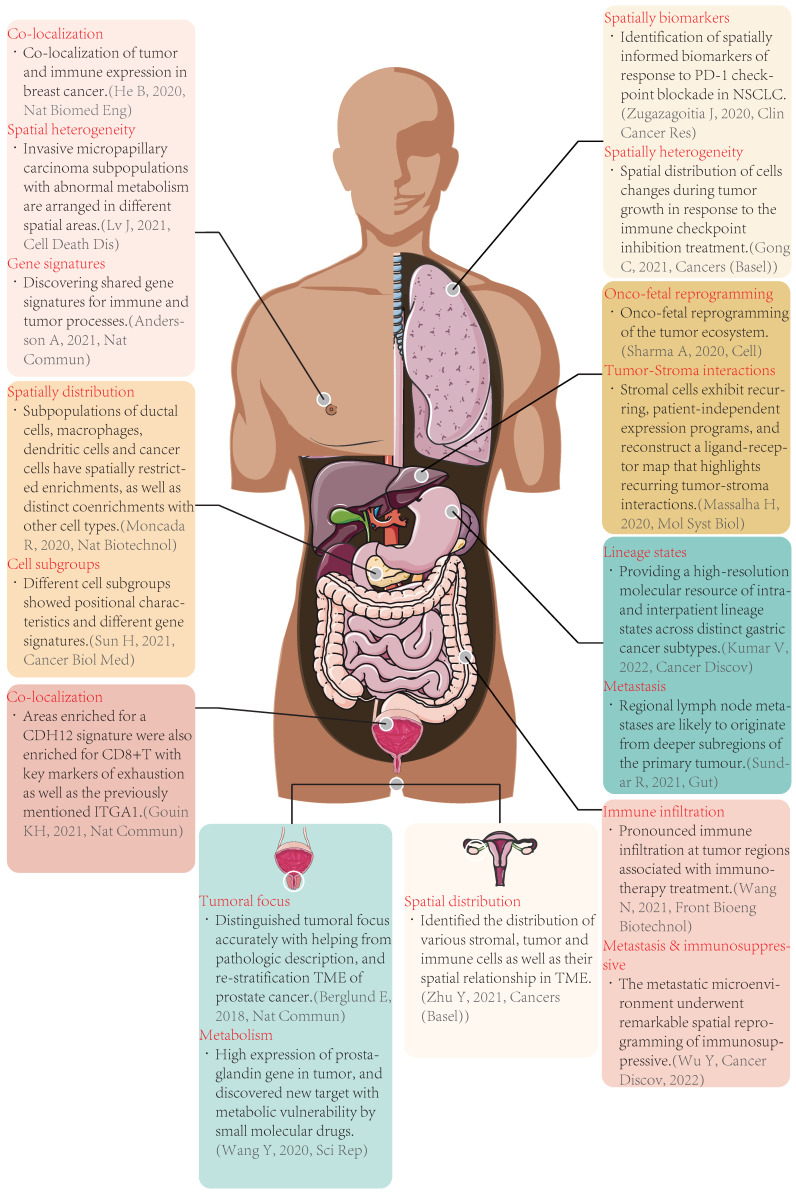
** Typical cases of spatial transcriptomics applied in tumors.** Nat Biomed Eng: Nature Biomedical Engineering; Nat Commun: Nature Communications; Nat Biotechnol: Nature Biotechnology; Cancer Biol Med: Cancer Biology & Medicine; Sci Rep: Scientific Reports; Clin Cancer Res: Clinical Cancer Research; Mol Syst Biol: Molecular Systems Biology; Cancer Discov: Cancer Discovery; Front Bioeng Biotechnol: Frontiers in Bioengineering and Biotechnology; CDH12: cadherin 12, type 2; CD8: cluster of differentiation 8; TME: tumor microenvironment; PD-1: programmed cell death protein 1; NSCLC: non-small cell lung cancer.

**Table 1:** Spatial transcriptomics in solid tumors

Author	Year	Journal	Type	Platform	Findings	Refs
**K. H. Gouin**	2021	Nat Commun	bladder cancer	Visium	The CDH12 population showed undifferentiated and aggressive	52
**Liu SQ**	2022	J Hematol Oncol	breast cancer	Visium	Malignant cells with diverse features, origins and functions clustered into distinct subpopulations owned specific spatial distribution and found variable enrichment of stromal cell subtypes	61
**Powell NR**	2022	Cancer Rep (Hoboken)	breast cancer	Visium	The heterogeneity of pharmacogenes expression may have significant connotations for cancer treatment because of the influence on drug distribution and efficacy	41
**S. Alon**	2021	Science	breast cancer	ExSeq	Differences in gene expression by cell types are plotted as a function of their distance from other cell types	62
**A. Andersson**	2021	Nat Commun	breast cancer	Visium	Found tertiary lymphoid-like structures, and colocalization of T-cell and macrophage subpopulation with type I interferon response	5
**A. Boisson**	2021	Front Mol Biosci	breast cancer	Visium	The tertiary lymphoid structure signature showed THF (two of the four annotated) and Th1 (all) expression	63
**B. He**	2020	Nat Biomed Eng	breast cancer	Visium	Identified spatially resolved gene expression data of 102 genes and the colocalization of tumor progression and activation of immune reaction	36
**R. Huang**	2020	Front Oncol	breast cancer	Visium	MAF positively regulated CD248 in the network of bone metastasis-specific	30
**P. Lu**	2021	Breast Cancer Res	breast cancer	Smart-3SEQ	Key transcriptomic diversity and copy number variations events preceding HER2 amplification in ductal carcinoma *in situ*	24
**J. Lv**	2021	Cell Death Dis	breast cancer	Visium	Drew the transcriptomic maps of IMPC and revealed its extensive heterogeneity, and found varieties programs of gene expression in stroma	21
**S. Nagasawa**	2021	Commun Biol	breast cancer	Visium	GATA3 irregulating promoted the transition of epithelial-to-mesenchymal and upregulated angiogenesis	28
**A. Kulasinghe**	2021	Front Oncol	breast cancer	DSP	Adjuvant chemotherapy response is related to the upregulation of ER-alpha and downregulation of CD137 and MART1 in the stroma	64
**J. Svedlund**	2019	EBioMedicine	breast cancer	ISS	Revealed intratumoral heterogeneity and uncovered areas of minor cellular subpopulations	65
**S. Vickovic**	2019	Nat Methods	breast cancer	HDST	High-resolution spatial transcriptomics developed to capture RNA on dense arrays of spatially barcoded beads from tissue sections	66
**L. Voith von Voithenberg**	2021	Small	breast cancer	DSP	Polyclonal origin of metastasis and development are driven by multiple positional specificities	29
**Y. Wang**	2021	Cancer Res	breast cancer	DSP	Expression morphology is an effective way to anticipate the mean expression of the tumor with spatial distribution from histology images	67
**S. Z. Wu**	2021	Nat Genet	breast cancer	Visium	High-resolution immune profiles provided by immunophenotyping, including a clinical associated new subpopulation of PD-L1/PD-L2(+) macrophage	16
**N. Yoosuf**	2020	Breast Cancer Res	breast cancer	ST	Trained a machine learning method on ST signatures and proved to be applicable to the identification of breast cancer regions	37
**V. Kumar**	2021	Cancer Discov.	gastrointestinal cancer	DSP	Showed molecular resources of lineage status within and between patients with different gastric cancer subtypes with high-resolution	46
**R. Sundar**	2021	Gut	gastrointestinal cancer	DSP	Regional lymph node metastases were related to deeper regions of originated tumor in gastric cancer	31
**N. Wang**	2021	Front Bioeng Biotechnol	gastrointestinal cancer	DSP	Compared with chemotherapy patients, the levels of PD-L1 expression in the tumoral region were increased in immunotherapy patients	25
**Y. Wu**	2021	Cancer Discov.	gastrointestinal cancer	Visium	Immunosuppressive cells showed significant spatially reprogramming in the metastatic microenvironment	6
**Qi J**	2022	Nat Commun	gastrointestinal cancer	Visium	FAP+ fibroblasts interact with SPP1+ macrophages to regulate the adherent proliferative microenvironment and limit immune cell infiltration into the tumor core	68
**L. A. Van de Velde**	2021	Cancer Res	glioma	Visium	Demonstrated a pathway by which arginine metabolizing myeloid cells and CD4(+) T cells synergize with pathogens to facilitate tumorigenesis	20
**Ravi VM**	2022	Cancer Cell	glioblastoma	Visium	Showed five differnet spatial transcriptional programs with unique genomic changes and common transcriptomic features Revealed the bidirectional and unidirectional interactions between microenvironment and temporal and spatial variations of GBM transcription heterogeneity	34
**Wang YF**	2022	Theranostics	liver cancer	Visium	CCL15 enriched in the core region and participated in building an immunosuppressive microenvironment CCL15 and CD163 relating to a poor prognosis while CCL19 and CCL21 indicated a good prognosis	44
**Wu R**	2021	Sci Adv	liver cancer	Visium	The relationship between TME remodeling and tumor metastasis and the distribution of PROM1(+) and CD47(+) cancer stem cell Revealed tertiary lymphoid structures	43
**H. Massalha**	2020	Mol Syst Biol	liver cancer	LCMseq	In liver metastases, stromal cells exhibit a recurrent, specific expression program and reconstitute the ligand-receptor profile	19
**N. M. Muñoz**	2020	Commun Biol	liver cancer	DSP	Molecularly targeted photothermal ablation effectively modulates the progression of intratumor myeloid cells to cells with immunogenicity and reduces the systemic release of cytokines related to tumorigenesis	69
**A. Sharma**	2020	Cell	liver cancer	DSP	VEGF and NOTCH signaling pathways relating to onco-fetal reprogramming	45
**C. Gong**	2021	Cancers (Basel)	lung cancer	spQSP-IO	Developed spQSP-IO, to extend QSP spatially resolved agent-based models for immuno-oncology	70
**J. Zugazagoitia**	2020	Clin Cancer Res	lung cancer	DSP	Clinical outcomes were only associated with high expression of CD56 and CD4 in CD45 compartment	38
**H. B. Schiller**	2019	Am J Respir Cell Mol Biol	lung cancer	Slideseq; MERFISH	Allowed for molecular characterization with a depth of TME and cellular neighborhoods	71
**M. Larroquette**	2022	J Immunother Cancer	Lung cancer	DSP	High CD163+ cell infiltration tumors showed ITGAM, CD27, and CCL5 upregulation High CD163+ cells infiltrated tumors showed high expression of ITGAM, CD27 and CCL5 High interferon-γ signaling pathway gene expression and the M1 phenotype related to exceptional immunotherapy outcomes in high macrophage infiltration tumors	39
**P. Nieto**	2021	Genome Res	melanoama; breast cancer; oropharyngeal squamous cell carcinoma;	Visium	Applied SPOTlight, identified the colocalization of various cell types in TME including stromal cells, cancer cells and immune-related cells by the combination of spatial transcriptomics and scRNA-seq	18
**K. Thrane**	2018	Cancer Res	melanoma	ST	Uncovered the components of melanoma metastases with spatially resolved data, and showed distinct gene expression profiles	72
**T. Vu**	2022	Nat Commun	melanoma	Visium; DSP	Demonstrated the feasibility of detecting protein and mRNA in cancer cells in the meantime	73
**E. Zhao**	2021	Nat Biotechnol.	melanoma; breast cancer; ovarian cancer	Visium	Showed that undetectable tissue structures previously and identified heterogeneity that histology images were unable to uncover	74
**Stur E**	2022	iScience	ovarian cancer	Visium	The spatial distribution and especially the spatial interaction of cell clusters showed a relationship with the reaction to chemotherapy	75
**Y. Zhu**	2021	Cancers (Basel)	ovarian cancer	SIO	The SIO pipeline identified cell markers and interactions among TME by cell segmentation and recognition of distinct cell features	76
**Hwang WL**	2022	Nat Genet	pancreatic cancer	DSP	Discovered recurring expression patterns in fibroblasts and cancerous cells Discovered three multicellular colonies constituted by a variety of cell subtypes and named classical, squamoid-basaloid and treatment enriched	50
**Sun H**	2021	Cancer Biol Med	pancreatic cancer	Visium	Hypoxic circumstances promoted transcriptome variation in spatial in pancreatic ductal adenocarcinoma, and identified potential targets for future therapy	77
**M. Elosua-Bayes**	2021	Nucleic Acids Res	pancreatic cancer	SPOTlight	Distinguished the tumor infiltrated and normal area, and mapped the distribution of tumoral special and clinically associated immune cell states	78
**K. Young**	2021	Gut	pancreatic cancer	DSP	The MLP-1 subtype was strongly linked with increased expression levels of immune genes, poor outcomes and an outbreak of tumor evolution	49
**M. R. Farren**	2020	JCI Insight	pancreatic cancer	DSP	Immunologically relevant proteins in different regions of the tumor microenvironment showed a noticeable change in spatial distribution and expression condition Significant changes were found in the spatial distribution and expression of immune-related proteins in different regions of the TME	79
**R. Moncada**	2020	Nat Biotechnol	pancreatic cancer	Visium	Inflammatory fibroblasts colocalized with stress-response genes expressed cancer cells	17
**Q. Song**	2021	Brief Bioinform	pancreatic cancer	Visium	Introduced deconvoluting spatial transcriptomics data through graph-based convolutional networks (DSTG)	80
**Brady L**	2021	Nat Commun	prostate cancer	DSP	Regarding tumor phenotype, there showed an intrapatient analogy Metastatic prostate cancer with high androgen receptor activity showed high expression of B7-h3/CD276	55
**E. Berglund**	2018	Nat Commun	prostate cancer	ST	Gradients pf gene expression found in the stroma adjacent, allowing for relayering of the TME	26
**E. Chelebian**	2021	Cancers	prostate cancer	Visium	Morphological images had certain relations with the molecular spectrum, which can predict the spatial distribution of a single gene	81
**S. Friedrich**	2020	BMC Med Genomics	prostate cancer	Visium	Explored a new method named STfusion which could help the spatial resolved localized of fusion transcripts in single cell level	82
**Y. Wang**	2020	Sci Rep	prostate cancer	Visium	Found that the tumor had higher levels of prostaglandin metabolic-related transcriptomes than the adjacent tissue	22
**A. L. Ji**	2020	Cell	squamous cell carcinoma	Visium	Colocalization of Tregs and CD8+ T cells in the stroma of stroma and such potential immunosuppressive mechanisms were uncovered	7
**M. Tarabichi**	2021	Mol Cell Endocrinol	thyroid cancer	ST	Showed that in the epithelial areas of this PTC there was increasing VIM transcription, including areas without fibroblasts	83
**K. H. Hu**	2020	Nat Methods	N/A	FISSEQ	Showed a peripheral to central track of myeloid and T-cell development	84
**Y. Lee**	2021	Sci Adv	N/A	XYZeq	Determined local expression of tumor suppressor genes by mesenchymal stem cells, which changes with proximity to the tumor core	85
**A. Levy-Jurgenson**	2021	Bioinformatics.	N/A	HTA	Created tumor molecular maps and whole-image heterogeneity maps, and developed a heterogeneity index	86
**B. T. Grünwald**	2021	Cell	pancreatic cancer	N/A	Concurrent intratumoral subTME generated patient's individual phenotypes and calculated predictable heterogeneity that was closely related to the biology of malignancy	87
**S. Kumar**	2020	Bio Protoc	glioma	N/A	Developed a method to classify tumor cells based on their relative distance from blood vessels	88

**Abbreviations:** CDH12: Cadherin 12; TFH: T follicular helper cells; Th1: T helper cell 1; CD248: tumor endothelial marker-1; HER2: human epidermal growth factor receptor 2; GATA3: GATA binding protein 3; estrogen receptor alpha; CD137: Cluster of Differentiation 137; MART1: melanoma antigen recognized by T-cells 1; PD-L1/PD-L2: programmed death-ligand 1/ programmed death-ligand 2; FAP: fibroblast activation protein; SPP1: secreted phosphoprotein 1; CD4: cluster of differentiation 4; CCL15: chemokine (C-C motif) ligand 15; CD163: cluster of differentiation 163; CCL19: chemokine (C-C motif) ligand 19; CCL21: Chemokine (C-C motif) ligand 21; TME: tumor microenvironment; PROM1: prominin 1; CD47: cluster of differentiation 47; VEGF: vascular endothelial growth factor; spQSP-IO: spatial quantitative systems pharmacology for immuno-oncology; QSP: quantitative systems pharmacology; CD163: cluster of differentiation 163; CD56: cluster of differentiation 56; CD45: cluster of differentiation 45; ITGAM: integrin alpha M; CD27: cluster of differentiation 27; CCL5: chemokine (C-C motif) ligand 5; SIO: SpatioImageOmics; DSTG: deconvoluting spatial transcriptomics data through graph-based convolutional networks; B7-h3/CD276: B7 homolog 3/ cluster of differentiation 276; CD8: cluster of differentiation 8; PTC: papillary thyroid carcinoma; VIM: Vimentin.

## Abbreviations

IR: infrared ray
UV: ultraviolet ray
scRNA-seq: single-cell RNA sequencing
NGS: next-generation sequencing
ST: spatial transcriptomics
ISH: *in situ* hybridization
FISH: fluorescent *in situ* hybridization
WM ISH: whole-mount ISH
LCM: laser capture microdissection
smFISH: single molecular FISH
ISS: *in situ* sequencing
DSP: GeoMX digital spatial profiler
MERFISH: multiplexed error-robust FISH
FISSEQ: fluorescent *in situ* sequencing
DBiT-seq: deterministic barcoding in tissue for spatial omics sequencing
COVID: corona virus disease
MHC: major Histocompatibility Complex
APOE: apolipoprotein E
TRBC1: T-Cell Receptor Beta Constant 1
HLA: human leukocyte antigen
ERBB2: Erb-B2 Receptor Tyrosine Kinase 2
EPCAM: epithelial cell adhesion molecule
CDH1: Cadherin 1
CD4: cluster of differentiation 4
IMPC: invasive micropapillary carcinoma
TME: tumor microenvironment
CNV: copy number variation
PD-1: programmed death-1
PD-L1: programmed death-ligand 1
DC: Dendritic cell
Tregs: regulatory T cells
MIBI-TOF: multiplexed ion beam imaging time-of-flight mass spectrometry
CNN: convolutional neural networks
NSCLC: non-small-cell lung cancer
CD163: cluster of differentiation 163
ITGAM: integrin alpha M
CD27: cluster of differentiation 27
CCL5: chemokine (C-C motif) ligand 5
CSF1R: colony stimulating factor 1 receptor
HER-2: human epidermal growth factor receptor 2
PROM1: prominin 1
CD47: cluster of differentiation 47
TLS: tertiary lymphoid structure
CCL15: chemokine (C-C motif) ligand 15
VEGF: vascular endothelial growth factor
MLP-1: metastasis-like primary
PDAC: pancreatic ductal adenocarcinoma
CTLA4: cytotoxic T-lymphocyte associated protein 4
B7-H3/CD276: B7 homolog 3/ cluster of differentiation 276
SCD1: stearoyl-CoA desaturase 1
SLCO2A1: solute carrier organic anion transporter family member 2A1
cDNA: complementary DNA
ROI: regions of interest
FFPE: formalin-fixed, paraffin-embedded
